# Anthropometric and Physical Fitness Differences Among Brazilian Adolescents who Practise Different Team Court Sports

**DOI:** 10.2478/hukin-2013-0008

**Published:** 2013-03-28

**Authors:** Diego Augusto Santos Silva, Edio Luiz Petroski, Adroaldo Cesar Araujo Gaya

**Affiliations:** 1 Federal University of Santa Catarina, Postgraduate Program in Physical Education, Center for Research in Human Performance and Kinanthropometry, Florianópolis, Brazil; 2 Federal University of Rio Grande do Sul, Porto Alegre, Brazil.

**Keywords:** sports performance, cross-sectional studies, anthropometric and fitness variables, youth, team court sports

## Abstract

The objective of this work was to compare the anthropometric and physical fitness characteristics of Brazilian adolescents who practise team court sports and to compare specific parameters obtained for adolescents with data from the general population. This was a cross-sectional study of 1,348 male adolescents grouped as follows: basketball players (n = 287), indoor soccer players (n = 665), handball players (n = 108) and volleyball players (n = 288), all between 10 and 14 years of age. Anthropometric (body mass, body height, arm span, and body mass index) and physical fitness data (flexibility, muscular strength, explosive power, speed, aerobic fitness and agility) were collected. The Brazilian population was used as a reference and compared to the adolescent subjects using Z scores for all variables. Anthropometric characteristics and performances in physical fitness tests differed (p<0.05) among players of different sports. In addition, for each variable assessed, adolescents who practised team court sports showed similar or improved results compared to their counterparts in the general population (p<0.05). Furthermore, the anthropometric and physical fitness characteristics differed depending on the team court sport practised. These findings may elucidate which physical abilities are most impacted by the practise of a particular team sport as well as help teachers and physical education and sport professionals identify talented adolescents.

## Introduction

Team court sports are characterised by intermittent activities, in which intensive efforts are carried out over short time periods that alternate with periods of low intensity. This intermittent feature requires the use of all three energy systems (aerobic, lactic anaerobic and alactic anaerobic) to meet the players’ metabolic demands. Moreover, these sports have complex demands that require a combination of individual skills, teamwork, technique, tactics and strategies, which contribute to the physical conditions of the players as well as the dynamic nature of team court sports, in general (Stone and Kilding, 2009).

In Brazil, the team court sports most practised by adolescents are basketball, indoor soccer, handball and volleyball (Azevedo et al., 2007). Generally, such sports are practised in schools for recreational, social, competitive and other purposes.

Basketball has become one of the most popular court sports in many countries. Paiva Neto and Cesar (2005) performed a literature review on the physical abilities required to play basketball and defined it as a high-intensity sport with significant physical contact, high speed and constant jumps and shifts (both to attack and to defend). As a result, the main features of general physical fitness involved in basketball are anaerobic endurance and speed of movement. Other authors have added agility as a key factor in this sport (Ziv and Lidor, 2009).

Indoor soccer is considered an indoor version of field soccer and is played with five athletes per team. The different types of displacement, including acceleration, kicking, passing, dribbling, tackling and jumping, likely resulting in significant neuromuscular adaptations that improve physical abilities such as agility, power, muscular strength and aerobic fitness (Alvarez et al., 2009; Castagna et al., 2007). Castagna et al. (2007) reported that, on average, adolescents who play indoor soccer reach maximal heart rates of 84% ± 5.4% and peak oxygen uptake rates (VO_2_) of 75% ± 11.2% during a game.

Handball is a sport with great anaerobic demand. During the game, tasks such as pushing and blocking require high power and strength levels in the limbs and trunk regions (Gorostiaga et al., 2005; Izquierdo et al., 2002; Wallace and Cardinale, 1997). Gorostiaga et al. (2005) reported that stronger players with higher body mass have an advantage in handball because the requirements of the game, such as throwing the ball with power and speed, are met through jumping and physical contact with the opponent.

The characteristics of volleyball, including speed, jumping for spikes and blocks at high intensities over a short period of time result in fast and agile athletes who possess a high level of muscular strength and aerobic fitness (Gabbett et al., 2008). Adolescents are selected for this sport based on their skills, performance levels, physique and muscular strength (Benetti et al., 2005).

During adolescence, the impact of any sports discipline on anthropometric and physical fitness variables may be masked by hormonal changes caused by general physical growth (Silva and Cabral de Oliveira, 2010; Pearson et al., 2006). While team court sports have been widely researched, no studies have been conducted comparing data from young athletes with the general population. Moreover, the differences in anthropometric and physical fitness characteristics among adolescents who practise the four most widely practised court sports in Brazil are also unknown. The present extensive study may elucidate which physical characteristics are most impacted by participation in a particular team sport as well as assist teachers and physical education (PE) and sport professionals in identifying talented individuals.

Based on research in other countries and the characteristics specific to each sport discipline, the hypotheses of this study are as follows: 1) basketball and handball athletes have more muscle mass than players of other team court sports because there is greater contact between athletes in these sports disciplines; 2) youth subjects who practise basketball and volleyball have higher strength in the lower limbs because they jump more often than players of other sports; 3) indoor soccer athletes exhibit greater flexibility in the sit and reach test due to their higher mobilisation of back and posterior thigh joints, which are in constant use during matches; in addition, due to characteristics specific to indoor soccer, these athletes are faster and have better aerobic endurance than those of other sports; 4) youth team court athletes have better physical fitness results than their youth counterparts from the general population.

Thus, this study has the following objectives: 1) to compare the anthropometric and physical fitness characteristics across Brazilian adolescents who practise team court sports and 2) to compare specific variables (anthropometric and physical fitness characteristics) of adolescents who participate in team court sports with data from a matched general population.

## Methods

For this cross-sectional study, data were extracted from the Brazil Sports Project (Projeto Esporte Brasil - PROESP-BR) from the National Secretariat for High-Performance Sports of the Ministry of Sports. More detailed information on the design and methodological aspects of the PROESP-BR have been previously published (Brazil Sports Project, 2007). The project was approved by the Ethics Committee on Human Research of the Federal University of Santa Catarina (UFSC), Florianópolis (SC), Brazil (Process number - 218/08).

The population studied consisted of male Brazilian students from 10 to 14 years of age enrolled in public and private schools. Sample selection was performed in a non-probabilistic intentional way; therefore, the PROESP-BR was disclosed and physical education teachers had the option of joining the project, assessing students and forwarding data to the PROESP-BR coordination.

During the 2004/2005 academic year, information on approximately 9032 male students (from 10 to 14 years of age) from 23 states of Brazil plus the Federal District was added to the PROESP-BR database. Data were collected in three states from the Midwestern Region (n = 1354), the Federal District (n = 469), eight states from the North-eastern Region (n = 1484), five states from the Northern region (n = 925), four states from the South-eastern region (n = 3374), and three states from the Southern Region (n = 1426). Of the students evaluated, 97.4% were studying in schools located in urban areas.

Participants in this study were required to fulfil the following inclusion criteria: 1) in-school participation (practising and competing) in basketball, handball, indoor soccer or volleyball for at least six months; 2) training sessions of at least 50 minutes and 3) 3 or more days per week of sports practise. Information about the sports discipline, practise time, duration and weekly frequency was obtained from teachers and submitted the data to PROESP-BR.

Anthropometric measurements (body mass, body height and arm span) and physical fitness tests were obtained by PE teachers from each school who joined the PROESP-BR. All teachers were trained and had access to instructions for the application of tests and measurements through an internet site that included a video for standardisation and visual presentation of measurement techniques (Brazil Sports Project, 2007) prepared by members of the School of Physical Education, Federal University of Rio Grande do Sul (UFRGS), Porto Alegre, Brazil.

Body mass was determined using a digital anthropometric scale calibrated from 0 to 150 kg with accuracy of 0.05 kg, and body height was measured using a portable stadiometer (fixed to the wall) calibrated from 0 to 200 cm with accuracy of 0.2 cm (Brazil Sports Project, 2007). For the measurement of body mass and height, adolescents removed their shoes and were instructed to wear a minimal amount of clothing (trunks and shirt). The body mass index (BMI) was calculated by dividing the body mass in kilograms by the squared height in metres. The arm span was determined by means of a tape measure (attached to the wall and parallel to the ground) with precision of 0.2 cm (Brazil Sports Project, 2007); the student was placed facing the wall for the measurement.

Standardisation and procedures for the physical fitness tests (flexibility, muscular strength, explosive power, speed, aerobic fitness and agility) were taken from the PROESP-BR (Brazil Sports Project, 2007) (Table 1).

Descriptive statistics regarding absolute and relative frequencies, mean values and confidence intervals were used to characterise the sample and physical tests performed. Data normality was verified and confirmed by the Kolmogorov-Smirnov test. The interaction between sport disciplines, anthropometric variables and physical fitness tests was verified. Since there was no interaction between variables (*p*>*0.05*), analysis of covariance (ANCOVA) was used to compare the anthropometric variables and physical fitness tests of adolescents from the four types of sports, using age (controlled variable) as the co-variable. The Bonferroni multiple comparison test was used to identify differences between the four of sports.

To compare the anthropometric and physical fitness variables of adolescents in this study with reference populations, the Z scores of all variables were calculated using the formula: Z = (X - M)/SD, where Z = Z score, X = raw score of the variable, M = mean of the variable in the reference population and SD = standard deviation of the variable in the reference population. The Z score was adjusted for age by ANCOVA. The Brazilian population was used as the reference for body mass, height and BMI, according to data previously published in a Brazilian study that investigated adolescents from different regions (Silva et al., 2010). The Brazilian population was also used as the reference for physical fitness tests, according to data reported by PROESP-BR (Brazil Sports Project, 2007). There are no data for Brazilian adolescents for variable arm span, which impairs the comparison of this study with the general population of young people in Brazil. The Bonferroni multiple comparison test and ANCOVA were applied at a significance level of 5%.

## Results

Of the 9,032 records of male adolescents aged between 10 to 14 years, 1,348 adolescents were practising one of the four sports (basketball, handball, indoor soccer, or volleyball), and these individuals composed the study sample. The adolescents had an average age of 12.3 ± 1.3 years, and basketball, indoor soccer, handball and volleyball players had an average age of 12.4 ± 1.4, 12.2 ± 1.3, 12.5 ± 1.2 and 12.5 ± 1.3 years, respectively.

Most adolescents (49.3%) played indoor soccer, and a minority played handball (8.0%). Table 2 shows the sample distributions, mean values and confidence intervals for each anthropometric and physical fitness variable analysed.

Regarding the anthropometric variables (Table 3), adolescents who played indoor soccer were lighter than those who played other sports (*p*<0.01), while adolescents who played basketball were taller than those who played other sports (*p*<0.01). Adolescents who played indoor soccer had lower BMI values than those who played basketball and volleyball (*p*<0.01). In addition, those who played basketball, handball and volleyball had greater arm spans than those who played indoor soccer (*p*<0.01).

For the physical fitness tests (Table 3), those who played indoor soccer and volleyball had a better performances in the flexibility test than those who played basketball (*p*<0.01). In the abdominal flexion test, basketball and indoor soccer players made more repetitions than volleyball players (*p*<0.01). In the upper limb strength test, those who played basketball had better performances than those who played other sports (*p*<0.01).

Moreover, adolescents who played basketball had longer standing long jump results than those who played indoor soccer and handball (*p* = 0.04). Adolescents who played indoor soccer covered greater distances in the 9-minute run test than those who played basketball and volleyball (*p*<0.01). The worst performances in the 20-m speed test were among those who played volleyball (*p* = 0.02). There were no significant differences in the performances of adolescents in the agility test (*p* = 0.14).

**Figure 1:**
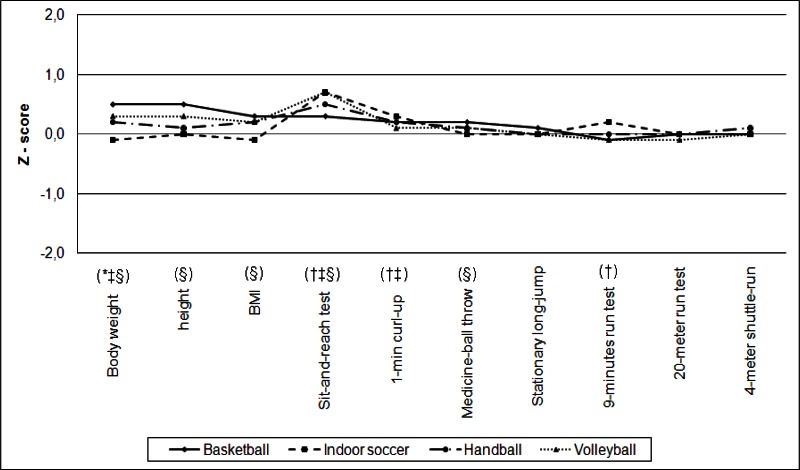
Z-scores of anthropometric variables and physical fitness tests of young athletes compared with the reference population ^*^Significant difference compared to Basketball players (p <0.05); ^†^*Significant difference in relation to Indoor soccer players (p <0.05);* ^‡^*Significant difference in relation to Handball players (p <0.05);* ^§^*Significant difference in relation to Volleyball players (p <0.05).*

Comparing the Z scores of specific parameters (anthropometric and physical fitness characteristics) of adolescents with data from the reference population (Figure 1) revealed that young athletes had similar values (Z score = 0) or values that were better than the general population (Z score > 0). Teenage basketball players showed higher values of anthropometric variables (body mass, body height, BMI) and better performances on tests of flexibility, abdominal strength, and explosive power (of upper and lower limbs). In turn, adolescents who played indoor soccer performed better than the reference population in flexibility, abdominal strength and aerobic fitness tests. Adolescents who played handball had higher body mass values and better performances in flexibility and abdominal strength tests. Adolescent volleyball players had higher values of anthropometric variables (body mass, body height, BMI) and better performances than the reference population on tests of flexibility and explosive power of upper limbs.

## Discussion

The most recent studies related to team sports report a complexity of physical characteristics inherent to their practitioners, and one of the most important are anthropometric variables. In this study, basketball, handball and volleyball players showed higher body mass and arm span values compared to the general population and indoor soccer players. Generally, practitioners of sport disciplines that require jumping and throwing with the upper limbs are taller, heavier and larger (Bayios et al., 2006; Drinkwater et al., 2007; Withers et al., 1997; Ziv and Lidor, 2009). Bayios et al. (2006) compared the anthropometric characteristics and body compositions of basketball and handball athletes, reporting that basketball players were taller than handball players. Withers et al. (1997) investigated the anthropometric characteristics of basketball, hockey and soccer players and found that basketball players were taller and heavier, thus presenting greater muscle mass, than players of other sports.

Information regarding the body height of the adolescents examined in this study should be interpreted with caution. Body height is not generally linked to sport specialisation and/or any sport in general because little or no change in body height can be accomplished through participation in sports. Moreover, not all adolescents in the present study preferred the same sports; some likely practised the only sports available at their school. Nonetheless, the authors of this study decided to keep body height as a descriptive variable in the analysis.

In this study, indoor soccer and volleyball players showed better performances in the flexibility test than basketball players. However, regardless of their sport disciplines, the performances of young athletes were, on average, 0.3 to 0.7 standard deviations above the mean reference population. In any sport, flexibility is essential for good performance. A possible explanation for the findings of this study is that flexibility is significantly affected by the movement autonomy to which the joint is regularly subjected (Erlandson et al., 2008). Young athletes exercise more than the general population, which can result in improved flexibility as well as other physical abilities. Moreover, the test employed in the present study requires flexibility in the dorsal and posterior thigh regions; thus, indoor soccer players, who use their legs constantly in the actions of the game, should rationally have an advantage.

In the abdominal strength test, basketball and indoor soccer players had better performance than those of other sport disciplines. Moreover, basketball, indoor soccer and handball players had 0.2, 0.3 and 0.2 standard deviations, respectively, above the collective performance score of the general population (*p* <*0.05*). During puberty, muscle strength is directly proportional to body height, such that taller adolescents tend to have higher muscle strength (Oliveira and Gallagher, 1997); this may explain our findings with regard to basketball players. Trainability is also expected to affect abdominal strength test results (Oliveira and Gallagher, 1997). Unlike volleyball, basketball, indoor soccer and handball require trunk mobility for the performance of dribbling; this difference in play may explain the similarity between volleyball players and the general population.

The literature reports that explosive power is an important feature for basketball players (Aşçi and Açikada, 2007; Paiva and César, 2005). In the present study, adolescents who practised basketball had better results in tests pertaining to explosive power than those who played other sports as well as the reference population. Ackland et al. (1997) reported that, on average, a basketball athlete jumps 46 times per game; this action should enhance basketball players’ performance scores when testing explosive power of the lower limbs.

In the present study, adolescents who played indoor soccer performed best in aerobic fitness tests compared to those who played other sports as well as the general population. Aerobic fitness is required for a player to perform well during an indoor soccer game because a high level of aerobic fitness decreases the probability of reaching fatigue (Alvarez et al., 2009; Castagna et al., 2009). Castagna et al. (2009) investigated the physiological demands during an indoor soccer game and reported high oxygen uptake rates and heart rates, indicating that aerobic fitness is a predominant requirement for success in indoor soccer. Alvarez et al. (2009) assessed the aerobic fitness of indoor soccer players at different competition levels and reported that these players had higher maximum oxygen uptake levels, better running economies and higher ventilatory thresholds than athletes who played other team sports at the same level.

The present study assessed the speed of the adolescents using the sprint test, which measures maximum speed that can be applied to any movement and depends on the development of agility, dynamic force, muscle elasticity, movement frequency and coordination as well as the domains of the movements employed. In the current study, basketball, indoor soccer and handball players performed better in this test compared to volleyball players. Sports such as indoor soccer, basketball and handball have intermittent characteristics and employ sprint speeds during attack and counterattack actions in the games, whereas volleyball generally utilises reaction speed (Castagna et al., 2009; Gabbett and Georgieff, 2007).

The present study has the following limitations: 1) the physical fitness tests employed were not specific to the sport disciplines investigated; however, because the disciplines differ in their particular technical characteristics, motor tests used in a physical fitness battery to detect new talents, such as the PROESP-BR, can yield useful results for comparisons between sports; 2) the cross-sectional design used in this study does not allow for the determination of cause/effect relationships (i.e., whether the adolescents had the same physical fitness levels before practising particular sports); 3) this study does not consider the effects of biological maturation stages because this variable has been found to affect performance results in physical tests as well as the body compositions of adolescents (Bar-Or, 1995; Pearson et al., 2006); 4) the data used in this study were collected more than five years ago; however, the data were collected nationwide on adolescents from all regions of Brazil and provide the information required for comparison of athletes’ results with those of the general population.

Notably the number of subjects studied for each sport differs widely because the study is part of a survey of the entire territory of Brazil. The distribution of subjects among the sports studied reflects the relative popularity of these sports in Brazil. Furthermore, the distribution found in this study is similar to previous published reports (Azevedo et al., 2007).

The present study is important for many reasons. First, the study employed data from all Brazilian regions, data that are difficult to obtain in a country with such diverse continental dimensions. Second the study included adolescents engaged in only one sports discipline at least three times per week, which greatly diminishes (or eliminates) the potential effects of other sports on their physical abilities. Third, this is the first study in Brazil to compare the anthropometric characteristics and physical fitness of adolescents practising the four most popular team court sports in Brazilian schools. Finally, this study compared young athletes to the normal population, which is important because, during adolescence, young people undergo biological maturation that can influence physical performance. Thus, this study concludes that the practise of team sports makes young people more physically fit than the normal population.

## Conclusions

Generalisation of the results of the present study is limited since the sample representativeness is low because schools that participated in the PROESP-BR did so voluntarily. Thus, the findings reported herein can only be interpreted in terms of the Brazilian adolescents from schools that participated in the project.

Compared to adolescents practising other sports, adolescents who played indoor soccer were lighter and had higher scores in flexibility, abdominal flexion, speed and aerobic fitness tests. Adolescents who played basketball were the tallest and had greater arm span, higher abdominal flexion, upper and lower limb strength and speed. Boys who played handball scored higher in the arm span test. Youth subjects who played volleyball scored higher in the arm span and flexibility tests. In addition, adolescents who practised team court sports performed better than the general population in all the tests/variables investigated (flexibility, abdominal flexion, upper and lower limb strength and aerobic fitness).

The present study is the first one to compare adolescents who practise team court sports with the general population. As such, it should help physical education and sports teachers to identify talents and to understand the physical variables most affected by the practise of these sports during adolescence.

## Figures and Tables

**Table 1:** The main physical components and the procedures used in each physical fitness test administered

Physical fitness test	Procedure
Sit-and-reach (flexibility)	The test evaluates flexibility. Subjects are seated with their legs joined and outstretched. The soles of their feet are supported in a standardised wood box (Well Box). Through inflection of their trunks, the subjects reach (with their ring fingers, arms joined and hands superposed) as far as they can toward/upon the box; they perform this reaching movement 2 times, and the maximal distance is recorded. The accuracy of this measurement in the present study is to within 0.1 cm.
1-min sit-ups (muscular endurance)	The test evaluates abdominal muscular endurance. Each subject is encouraged to do as many repetitions as they can in 1 minute (n =1).
Medicine-ball throw (power of upper limbs)	The test evaluates the power of the upper limbs. Each subject is seated with the backside of his trunk touching a wall. He then holds a medicine-ball with his hands (abreast of his chest) and throws it ahead as far as he can. He performs this action 2 times, and the maximal distance is recorded. The accuracy of this measurement in the present study is to within 0.1 cm.
Standing long-jump (power of lower limbs)	The test evaluates the power of the lower limbs. While standing, the subject propels himself by inflecting his knees jumping forward as far as he is capable. He performs this action 2 times, and the maximal distance is recorded. The accuracy of this measurement in the present study is to within 0.1 cm.
9-minute run (cardiorespiratory fitness)	The test evaluates cardio-respiratory fitness. Subjects run as far as they can in 9 minutes (n = 1). The accuracy of this measurement in the present study is to within 0.1 m.
20 m sprint (speed)	The test evaluates running speed. From a standing start, the subjects run for 20 metres. They do this 2 times, and the best time recorded. The accuracy of this measurement in the present study is to within 0.01 s.
4 m shuttle-run (agility and coordination)	The test evaluates agility and coordination. Subjects shift, as quickly as they are able, in a square area. They do this 2 times, and the best time is recorded. The accuracy of this measurement in the present study is to within 0.01 s.

**Table 2:** Sample distribution(s) according to the sport discipline practised and descriptive values of anthropometric variables and physical fitness tests

Variables	n	%	(CI 95%)
*Total*	1348	100	

Basketball	287	21.3	(19.1 – 23.5)
Indoor soccer	665	49.3	(46.7 – 52.0)
Handball	108	8.0	(6.5 – 9.5)
Volleyball	288	21.4	(19.2 – 23.6)

	n	M	(CI 95%)

Age (years)	1348	12.3	(12.2 – 12.4)
Body mass (kg)	1348	46.3	(45.6 – 46.9)
Body height (cm)	1348	156.3	(155.6 – 157.0)
BMI (kg/m^2^)	1348	18.6	(18.5 – 18.8)
Arm span (cm)	1346	159.6	(158.7 – 160.4)
Sit-and-reach test (cm)	1327	24.7	(24.2 – 25.2)
1-min sit-ups (repetitions)	1343	33.6	(33.1 – 34.0)
Medicine-ball throw (cm)	1342	316.0	(311 – 322)
Stationary long-jump (cm)	1345	160.7	(159.1 – 162.3)
9 min run test (m)	1229	1436.7	(1416.9 – 1456.4)
20 m run test (s)	1342	3.8	(3.7 – 3.9)
4 m shuttle-run (s)	1342	6.5	(6.4 – 6.5)

CI: confidence interval, M: mean, BMI: body mass index

**Table 3:** Analyses of covariance (co-variable = age) comparing mean values and confidence intervals of anthropometric variables and physical fitness according to sports discipline

Variable	Basketball	Indoor soccer	Handball	Volleyball	F	*p*

	M (CI 95%)	M (CI 95%)	M (CI 95%)	M (CI 95%)		
Body mass (kg)	50.0 (48.7–51.2)^†^	43. 7 (42.9–44.5)	47.0 (45.0–49.0)^†^	47.9 (46.7–49.1)^†^	6.7	<0.01
Body height (cm)	159.5 (158.5–160.5)^†‡§^	154.2 (153.6–154.9)	156.3 (154.6–157.9)	157.2 (156.2–158.2)^†^	8.7	<0.01
BMI (kg/m^2^)	19.3 (18.9–19.6)^†^	18.1 (17.8–18.3)	18.9 (18.3–19.5)	19.1 (18.7–19.4)^†^	13.1	<0.01
Arm span (cm)	163.5 (162.1–164.9)^†^	156.8 (155.8–157.6)	160.8 (158.4–163.1)^†^	161.2 (159.8–162.7)^†^	4.2	<0.01
Sit-and-reach test (cm)	22.2 (21.1–23.2)	25.6 (24.9–26.3)^*^	24.1 (22.3–25.7)	25.5 (24.4–26.5)^*^	11.3	<0.01
1-min sit-ups (repetitions)	33.8 (32.8–34.8)^§^	34.0 (33.3–34.6)^§^	33.2 (31.5–34.9)	31.8 (30.7–32.7)	10.2	<0.01
Medicine-ball throw (cm)	324.9 (317.8–331.9)^†‡§^	298.6 (294.5–302.6)	294.3 (284.2–304.4)	304.3 (296.7–311.8)	14.6	<0.01
Stationary long-jump (cm)	166.0 (163.1–168.9)^†‡^	157.9 (156.0–159.8)	157.0 (152.2–161.8)	162.5 (159.6–165.5)	2.6	0.04
9 min run test (m)	1374.4 (1332.9–1415.9)	1486.5 (1459.5–1513.5)^*§^	1430.0 (1358.6–1501.3)	1379.9 (1338.6–1421.1)	9.7	<0.01
20 m run test (s)	3.8 (3.7–3.8)^§^	3.8 (3.7–3.8)^§^	3.8 (3.7–3.9)	4.0 (3.9–4.0)	5.1	0.02
4 m shuttle-run (s)	6.5 (6.4–6.6)	6.4 (6.3–6.5)	6.6 (6.5–6.8)	6.5 (6.4–6.6)	1.8	0.14

M: Mean, CI: confidence interval, F: analysis of covariance value.

* Significant difference compared to Basketball players (p < 0.05);

† Significant difference in relation to Indoor soccer players (p < 0.05);

‡ Significant difference in relation to Handball players (p < 0.05);

§ Significant difference in relation to Volleyball players (p < 0.05)
